# Eosinophilia and potential antibody cross-reactivity between parasites in a child with pinworm and immune dysregulation: a case report

**DOI:** 10.1186/s12887-023-04006-0

**Published:** 2023-04-27

**Authors:** Maria Di Cicco, Giulia Bertolucci, Carlotta Gerini, Fabrizio Bruschi, Diego G. Peroni

**Affiliations:** 1 Azienda Ospedaliero Universitaria Pisana – Pisa University Hospital, U.O. Pediatria – Pediatrics Unit, Via Roma n. 67 –, 56126 Pisa, Italy; 2 Department of Clinical and Experimental Medicine, University of Pisa, Pisa, Italy; 3 Department of Translational Research, N.T.M.S., University of Pisa, Pisa, Italy; 4 Programma Monitoraggio Parassitosi e f.a.d., Pisa University Hospital, Pisa, Italy

**Keywords:** Autoimmunity, Children, Enterobiasis, Enterobious vermicularis, Intestinal parasite infection

## Abstract

**Background:**

Intestinal parasitic infections are common in humans, especially among young children. These conditions are often asymptomatic and self-limiting, and diagnosis is mainly based on the search for ova and parasites in the stools since serology may be biased due to cross reactivity between parasites. Pinworm is common in children and is not usually associated with hypereosinophilia; adhesive-tape test is the gold standard testing for the microscopic detection of Enterobious vermicularis (Ev) eggs.

**Case presentation:**

A 13-year-old boy was referred due to a self-resolving episode of vomiting and palpebral oedema after dinner, together with a history of chronic rhinitis, chronic cough, absolute IgA deficiency and Hashimoto’s thyroiditis and hypereosinophilia (higher value = 3140/µl). On evaluation we detected only palpable thyroid and hypertrophic nasal turbinates. Food allergy was excluded, but skin prick tests showed sensitization to house dust mites and cat epithelium and spirometry showed a marked obstructive pattern with positive bronchodilation test prompting the diagnosis of asthma for which maintenance inhaled treatment was started. Chest x-ray and abdomen ultrasound were negative. Further blood testing showed positive IgG anti-Echinococcus spp. and Strongyloides stercoralis and positive IgE for Ascaris, while Ev were detected both by the adhesive tape test and stool examination, so that we made a final diagnosis of pinworm infection. Three months after adequate treatment with pyrantel pamoate the adhesive-tape test turned out negative and blood testing showed a normal eosinophil count. The child later developed also type 1 diabetes.

**Conclusions:**

We suggest the need to investigate for enterobiasis in children with hypereosinophilia and to consider autoimmunity as a potential confounding factor when interpreting serology for helminths.

## Background

Intestinal parasitic infections are common in humans, especially among young children, who have the habit of touching their mouths with their hands without washing them. These conditions are often asymptomatic and self-limiting, and diagnosis is mainly based on the search for ova and parasites in the stools; adhesive-tape test collected in early morning prior to washing the perianal area is the gold standard testing for the microscopic detection of *Enterobious vermicularis* (Ev) eggs [1]. Serology is often inconclusive and may be biased due to cross reactivity between parasites. It is commonly believed that Ev is not frequently associated with blood eosinophilia, especially in children. Here, we report the case of a pinworm infection in a 13-year-old boy with Hashimoto’s thyroiditis, absolute IgA deficiency, allergic asthma and severe hypereosinophilia, showing positive serology to several helminths. The child later developed also type 1 diabetes.

## Case presentation

A 13-year-old boy was referred in April 2021 to our Allergology section due to a self-resolving episode of vomiting and palpebral oedema after dinner. The same meal was taken in other subsequent occasions without him developing any symptoms, therefore we excluded food allergy. The boy had a history of chronic rhinitis, absolute IgA deficiency and Hashimoto’s thyroiditis since the age of 10 years and was on levothyroxine treatment. In 2018, hypereosinophilia had been reported for the first time (eosinophil count = 1600/µl), reaching the peak in July 2020 (blood cell count 9100/µl, eosinophil count = 3140/µl) but was considered secondary to suspected allergic rhinitis. In the previous year, the boy had also complained of dry cough with physical exertion and during the night. On evaluation the boy looked healthy, and we only detected palpable thyroid and hypertrophic nasal turbinates. Skin prick tests for aeroallergens were performed, demonstrating sensitization to house dust mites (HDM) and cat epithelium, while spirometry showed a marked obstructive pattern with positive bronchodilation test (FEV_1_ + 32%). FeNO was 32 ppb (normal values < 25). Therefore, we prescribed long-term treatment with inhaled fluticasone + salmeterol together with nasal mometasone. We also performed further blood tests to investigate hypereosinophilia, which confirmed such evidence (white blood cells = 10,600/µl, eosinophil count = 2010/µl; 19%) and demonstrated a marked increase in serum total IgE levels (4968 U/mL), undosable IgA, and normal serum protein levels, except for that of β1 globulin. A reduced number of CD8 + lymphocytes (17.1%, normal range = 20–28%) and a slight increase in the numbers of CD19 + lymphocytes (16.5%, normal range = 5–14%) with normal percentages of other lymphocyte subpopulations were also observed.

**Fig. 1:**
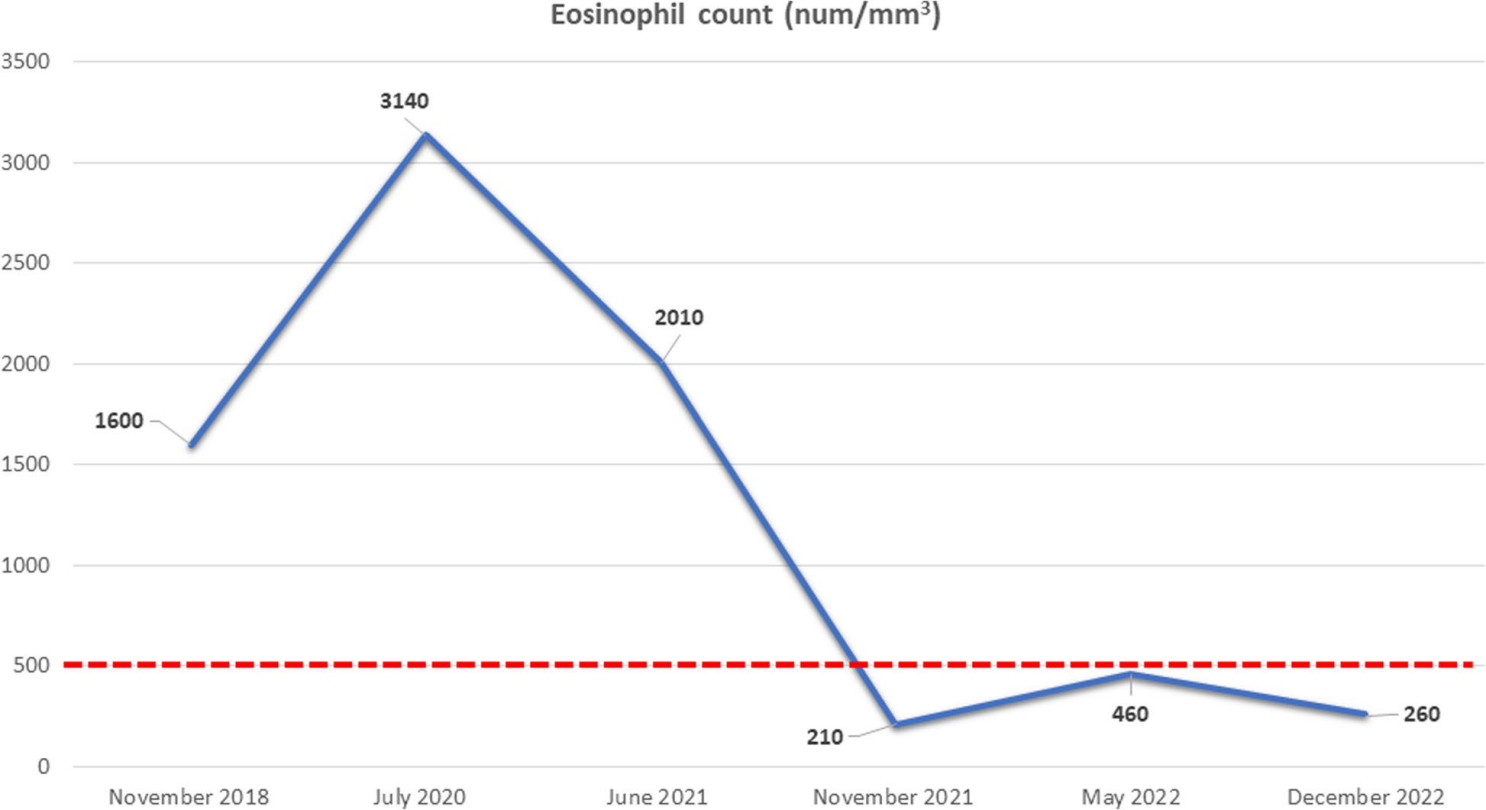
The figure shows eosinophils count trend; note the steep fall of eosinophils after Ev eradication

Positivity for specific IgG anti-*Echinococcus* spp. and *Strongyloides stercoralis* by ELISA commercial kits and positive IgE for *Ascaris* were also detected (64.2 KUA/L, normal values up to 0.1), while the search for antibodies against *Taenia solium*, *Toxocara* spp. and *Trichinella* spp. as well as the search for PR3-ANCA and MPO-ANCA were negative. Tropical causes of eosinophilia such as Wuchereria bancrofti infection, were not investigated considering their rarity in our country and the fact that the patient had never travelled abroad. Then, we performed chest x-rays and abdominal ultrasound, which were normal, while in the third sample, stool examination showed positivity for pinworms, which was confirmed by the adhesive-tape test. The boy later revealed that he had suffered for at least 2 years of perianal pruritus. We administered two doses of pyrantel pamoate, separated by 2 weeks, to all household members and, three months later, the adhesive-tape test was repeated and turned out negative. Spirometry showed an improvement of all ventilatory parameters with negative bronchodilator test and FeNO was normal (12 ppb). Blood testing showed a normal eosinophil count (Fig. 1) and persisting positive serology for *Strongyloides*, while Western blot (Wb) for *Echinococcus* was negative and IgE levels dropped to 2230 U/mL. Component-resolved diagnostics related to HDM allergy demonstrated sensitization to tropomyosin (Der p 10 IgE > 100 KUA/L). The patient and his parents were relieved by the efficacy of the treatment. Unfortunately, a couple of months later, the boy was admitted due to onset of type 1 diabetes mellitus; blood testing continued to show normal eosinophil count 6 and 12 months later. Notably, symptoms of Hashimoto’s thyroiditis as well as thyroid function tests were controlled since the start of levothyroxine treatment and during the entire period of follow up.

## Discussion and conclusions

Enterobiasis or pinworm is caused by cecum infection by *E vermicularis* (Ev) and represents the most common and the least pathogenic of all intestinal parasitic infections in both adults and children. Transmission occurs via faeco-oral route and infected individuals can transmit Ev eggs to others through contaminated hands, bedding, clothing and other surfaces, so that the infection usually spreads among children attending daycare and all the members of the household [2].

Most children with enterobiasis are asymptomatic, but many report intense nocturnal perianal itching, insomnia, restlessness, irritability and urticaria. Vulvovaginitis, weight loss, diarrhoea, recurrent abdominal pain, and colitis with eosinophilia have also been reported [2]. Ev localization in the appendix may mimick appendicitis.

Our case has several peculiarities which deserve to be reported. First of all, it should be noted that our patient had severe hypereosinophilia prompting laboratory blood testing, which is not routinely performed when a pinworm infection is suspected: further blood testing was performed to investigate parasite serology as well as ANCA levels, considering that our patient also had allergic asthma and chronic rhinitis which could have been related to a potential diagnosis of eosinophilic granulomatosis with polyangiitis. Regarding the eosinophil count, despite the well-established correlation between eosinophilia and other helminth infections (such as those caused by *Toxocara*, *Trichinella* species, *T. solium*, *Echinococcus*, *Strongyloides*), it is commonly believed that Ev is not frequently associated with blood eosinophilia, especially in children, but it should be noted that these patients are usually healthy enough at the first evaluation to avoid blood testing, which is why data is probably lacking on this point [2]. Unsurprisingly, in 2019 Shroeder et al. reported 3 cases of peripheral eosinophilia in children with pinworm and gastrointestinal complaints suggesting an association between Ev and high eosinophil count, but none of them had an eosinophil count higher than 1,500, as in our patient [3]. In the literature, there are only a few other reports confirming that eosinophilia may be associated with enterobiasis in adults. It should be noted that in our patient the eosinophil count significantly improved after treatment with pyrantel pamoate, confirming enterobiasis as the cause, perhaps due to a long-lasting infection with a large worm burden, which was confirmed by the fact that the stool examination was positive, while it commonly detects only 5% of Ev intestinal infestations. Moreover, it should be noted that while hypereosinophilia and high levels of IgE can be found in children with severe allergic asthma, especially with atopic dermatitis, our patient, despite having a long story of hypereosinophilia, showed mild asthma symptoms, a low increase of FeNO and optimal response to asthma treatment. Notably, inhaled corticosteroids have a limited effect on peripheral eosinophilia. Thus, in our opinion, Ev should be considered in the diagnostic algorithm for eosinophilia in children, even in the absence of typical symptoms, in order to avoid more aggressive and unproductive evaluation [4].

We also found positive serology for *Echinococcus* spp. and *S. stercoralis* in our patient; the former was later excluded by specific Wb testing, while the latter was persistent even after Ev eradication even if strongyloidiasis was excluded on the basis of stool examination. It should be considered that, while the sensitivity of serological assays for Strongyloides in non-endemic areas reaches 89% and the specificity is limited to 54% [5], many of the serological tests for helminths cross-react due to a high degree of molecular and structural similarities among helminth antigens (as an example, filiariasis and strongyloidiasis) [6]. In our case polyclonal antibody production may have been triggered by the presence of active immune dysregulation which already caused Hashimoto thyroiditis as well as asthma, and later determined the onset of type 1 diabetes. As a consequence, we plan to perform genetic testing to better evaluate immune functioning in this patient. Interestingly, false positive serological tests for *Borrelia burgdorferi* have been reported in a in a 32- year-old woman with thyroiditis [7]: such evidence supports the hypothesis that autoimmunity and immune dysregulation in general may interfere with serological diagnosis of infectious diseases. To our knowledge, our case is the first proposing potential multiple parasite cross-reactivity in the context of immune dysregulation with multiple clinical manifestations.

Moreover, the high level of IgE in our patient may have been determined by both allergy and Ev infection: a recent study by Patsantara et al. investigated the immune response in 215 children from Greece, of whom 105 were infected with Ev and 110 were matched healthy controls. The study reported higher levels of eosinophils, eosinophil cationic protein and total IgE in the infected children, indicating a type-2 immune response activation during infection, similarly to what happens in allergic children. Moreover, the atopic infected children exhibited higher IgE levels than the non-atopic ones [8].

Last but not least, our patient had positive IgE against *Ascaris lumbricoides*, which may be linked to HDM sensitization: as a matter of fact, intestinal helminths and arthropods such as *Dermatophagoides pteronyssinus* share a high degree of molecular and structural similarities which potentially generate cross-reacting antibodies. In particular, a high degree of structural homology between mite tropomyosins (Der p 10) and ascaris tropomyosin (Asc l 3) has been reported, and our patient was sensitized to Der p 10 [9]. Tropomyosins are considered pan-allergens and are highly conserved across many species, so that cross-reactivity is expected, and such antibodies may play a role in the pathogenesis or regulation of both allergy and parasite infection. Overall, whether helminth infections confer protection against or serve as risk to the development of allergic diseases and vice versa, and the mechanisms on how these occur is still a matter of debate [10]. It should be noted that a meta-analysis of 30 clinical studies demonstrated that *A. lumbricoides* infection was associated with aggravated asthma symptoms [11].

In conclusion, our case supports the need to investigate for Ev in children with hypereosniophilia and suggests that autoimmunity may be considered a confounding factor when interpreting serology for helminths.

## Data Availability

Data supporting the findings of this study are available from the corresponding author, Dr. Maria Di Cicco, upon reasonable request.

## Abbreviations

ANCA: Anti-Neutrophil Cytoplasmic Antibodies
Ev: Enterobius vermicularis
HDM: House Dust Mites
FEV1: Forced Expiratory Volume in one second
Wb: Western blot

## References

[1] Ricciardi A, Ndao M (2015). Diagnosis of parasitic infections: what’s going on?. J Biomol Screen.

[2] Pinto B, Bruschi F. Pinworm. Reference Module in Biomedical Sciences. Elsevier; 2021.

[3] Schroeder JC, Jones D, Maranich A (2019). Peripheral eosinophilia found in Pediatric Enterobius vermicularis infections. Clin Pediatr (Phila).

[4] Costagliola G, Di Marco S, Comberiati P, D’Elios S, Petashvili N, Di Cicco ME (2020). Practical Approach to Children presenting with Eosinophila and Hypereosinophilia. Curr Pediatr Rev.

[5] Kalantari N, Chehrazi M, Ghaffari S, Gorgani-Firouzjaee T (2020). Serological assays for the diagnosis of Strongyloides stercoralis infection: a systematic review and meta-analysis of diagnostic test accuracy. Trans R Soc Trop Med Hyg.

[6] Checkley AM, Chiodini PL, Dockrell DH, Bates I, Thwaites GE, Booth HL (2010). Eosinophilia in returning travellers and migrants from the tropics: UK recommendations for investigation and initial management. J Infect.

[7] Garment AR, Demopoulos BP (2010). False-positive seroreactivity to Borrelia burgdorferi in a patient with thyroiditis. Int J Infect Dis.

[8] Patsantara GG, Piperaki ET, Tzoumaka-Bakoula C, Kanariou MG (2016). Immune responses in children infected with the pinworm Enterobius vermicularis in central Greece. J Helminthol.

[9] Gazzinelli-Guimaraes PH, Bennuru S, de Queiroz Prado R, Ricciardi A, Sciurba J, Kupritz J (2021). House dust mite sensitization drives cross-reactive immune responses to homologous helminth proteins. PLoS Pathog.

[10] McSorley HJ, Chayé MAM, Smits HH (2019). Worms: pernicious parasites or allies against allergies?. Parasite Immunol.

[11] Leonardi-Bee J, Pritchard D, Britton J (2006). Asthma and current intestinal parasite infection: systematic review and meta-analysis. Am J Respir Crit Care Med.

